# A New Dental College

**Published:** 1893-05

**Authors:** 


### A New Dental College.

We see from the public prints that the incorporation for a third dental college in Cincinnati has been effected, with a capital of ten thousand dollars, with six incorporators. It does not appear whether the faculty has been appointed yet or not, or when or where the college will be established. When that is done Ohio will then have six dental colleges, and though not one-third as many as Chicago has, yet we think quite enough for the present need.

